# Clinical Significance of Lactate Dehydrogenase A Expression in Colorectal Cancer

**DOI:** 10.7759/cureus.98628

**Published:** 2025-12-07

**Authors:** Jarunya Ngamkham, Hathaiwan Moungthard, Thiwaporn Thesawadwomg, Maneerut Mus-U-Dee, Thainsang Phansri, Krittika Boonmark, Thitaporn Phukhan

**Affiliations:** 1 Department of Research and Technology Assessment, National Cancer Institute, Bangkok, THA; 2 Department of Gastrointestinal and Liver Clinic, National Cancer Institute, Bangkok, THA; 3 Department of Anatomical Pathology, National Cancer Institute, Bangkok, THA; 4 Division of Medical Policy and Strategy Development, Maha Vajiralongkorn Thanyaburi Hospital, Pathum Thani, THA

**Keywords:** colorectal cancer, gene expression, lactate dehydrogenase a, ldha, metabolic reprogramming

## Abstract

Introduction: Metabolic reprogramming, characterized by alterations in metabolic pathways to support energy production and biosynthesis, is a hallmark of cancer and is exemplified by the Warburg effect. This process plays a pivotal role in tumor progression and is evident across multiple cancer types, including colorectal cancer (CRC).

Objectives: This study aimed to investigate the expression profiles of metabolism-related genes in CRC cells at early and advanced stages and to validate the expression of selected genes in CRC tissue samples.

Methods: Tissues from patients with early and advanced stages of CRC were collected to investigate the expression profiles of metabolism-related genes using microarray analysis. Selected genes were further validated at both the mRNA and protein levels by reverse transcription polymerase chain reaction (RT-PCR) and immunohistochemistry (IHC), respectively. Based on gene profiling from microarray analysis, the metabolism-related gene lactate dehydrogenase A (LDHA) was selected for further investigation. A retrospective evaluation of LDHA expression was conducted using 260 formalin-fixed, paraffin-embedded (FFPE) tissue sections obtained from CRC patients diagnosed between January 2021 and December 2023 at the National Cancer Institute, Thailand.

Results: Comparative analysis revealed 758 upregulated and 619 downregulated pathways in early-stage CRC and 630 upregulated and 614 downregulated pathways in advanced-stage CRC. From all of these pathways, altered metabolism-related pathways were predominantly associated with glycolysis, oxidative phosphorylation, and CRC metabolic reprogramming. Among metabolism-related genes, LDHA exhibited significantly elevated expression in CRC tissues compared to adjacent normal tissues, with marked increases at both mRNA and protein levels in early and advanced stages. Statistical analysis demonstrated a significant correlation between LDHA expression and metastasis to lymph nodes as well as distant organs, including the lungs and liver.

Conclusions: These findings suggest that metabolic reprogramming is strongly linked to CRC progression and that LDHA may serve as a promising biomarker for CRC screening, prognosis, and therapeutic intervention.

## Introduction

Colorectal cancer (CRC) is the third most frequently diagnosed cancer worldwide and the second most leading cause of cancer-related mortality, with more than 1.9 million new cases and approximately 900,000 deaths reported in 2022 [1]. CRC typically develops slowly over several years and is often asymptomatic during the early stage, contributing to delayed diagnosis. The trends in CRC incidence rates have declined in older adults in the past few years, largely attributable to improved primary screening and early detection strategies. Conversely, a concerning increase in incidence rates has been observed among younger adults under 50 years of age [2,3]. Multiple risk factors contributing to CRC development were driven by lifestyle habits, genetic mutation, physical and chemical risk factors, obesity, environmental exposures, etc. [4]. CRC represents a highly heterogeneous disease with different molecular characteristics that influence diagnosis accuracy, treatment response, and clinical outcomes. Genetic alterations in oncogene and tumor suppressor genes, which are mostly found in KRAS, BRAF, Wnt, SMAD4, and p53, play a pivotal role in CRC development by disrupting cellular metabolism, proliferation, and differentiation [5,6]. 

Cellular metabolism encompasses a complex network of biochemical reactions that enable cells to convert nutrients into essential biomolecules, maintain cellular homeostasis, and generate energy in the form of adenosine triphosphate (ATP). In addition, metabolic pathways provide biosynthetic precursors, including amino acids, nucleotides, and fatty acids [7,8]. In cancer cells, these metabolic pathways are extensively reprogrammed by the increasing demand for rapid proliferation and survival. Cancer cells also achieve this by altering energy production and enhancing the synthesis of nucleotides, lipids, and proteins necessary for continuous growth and division [9,10]. This phenomenon, known as metabolic reprogramming, is a fundamental cancer hallmark and plays a crucial role during tumorigenesis and cancer progression. Cancer cells exhibit a high demand for oxygen and energy, leading them to preferentially utilize aerobic glycolysis rather than oxidative phosphorylation (OXPHOS), even under normoxic conditions. Although glycolysis is less efficient than OXPHOS in ATP generation, this metabolic shift provides a rapid source of energy and biosynthetic intermediates essential for anabolic growth [11-13]. This reprogrammed metabolic state, known as the "Warburg effect", is a defining feature of many malignancies, including CRC. In this context, cancer cells preferentially convert pyruvate to lactate through the action of lactate dehydrogenase (LDH) enzyme, thereby regenerating NAD⁺ and sustaining glycolytic flux, while mitochondrial OXPHOS is concomitantly reduced [9,12].

LDH is a key regulatory enzyme that catalyzes the final step of glycolysis, facilitating the interconversion between pyruvate and lactate. It plays an important role in metabolic reprogramming, particularly in promoting the Warburg effect, by supporting the energy and biosynthetic requirements of malignant cells [10,12,14]. LDH exists as two major isoenzymes, lactate dehydrogenase A (LDHA) and lactate dehydrogenase B (LDHB), that catalyze the same reversible reaction but differ in tissue distribution and metabolic preference. LDHA is predominantly expressed in cancer cells and favors the conversion of pyruvate to lactate, whereas LDHB is commonly found in non-cancerous cells to promote the reverse reaction from lactate to pyruvate [15]. Recent studies have reported that LDHA is markedly highly expressed in various types of cancer and strongly associated with poor clinical prognosis and treatment outcome. Overexpression of LDHA has been linked to chemotherapy resistance through the maintenance of cancer stem cell (CSC) stemness, mediated by enhanced lactate production and the establishment of an acidic tumor microenvironment [16]. Furthermore, elevated LDHA expression correlates significantly with tumor size, clinical stage, and histological grade and is inversely associated with disease-free survival and overall survival in clear cell renal cell carcinoma patients [17]. Inhibition of LDHA expression has been shown to suppress cancer cell proliferation, tumorigenesis, and progression, reduce the expression of CSC markers, and promote apoptosis in lung and colon cancers [18-20]. Moreover, LDHA knockdown attenuates the progression of CSCs and drug-resistant cancer phenotypes driven by oncogenic KRAS and/or EGFR signaling pathways [20-21]. Collectively, these findings underscore the pivotal role of LDHA in cancer metabolism, tumor progression, and therapeutic resistance, with potential as a prognostic biomarker and therapeutic target.

The purposes of this study were to investigate the expression profiles of metabolism-related genes, to validate the mRNA and protein expression levels of LDHA, a key regulatory enzyme in the Warburg effect, in CRC tissues at early and advanced stages, and to assess their prognostic significance. The expression levels of LDHA mRNA and protein may improve the accuracy of CRC prediction across both early and advanced stages. In addition, overexpression of LDHA was correlated with the advanced stage of CRC and clinical parameters of metastasis, such as lymph node invasion. Gene expression profiling was performed using microarray analysis, followed by the validation of LDHA expression at the protein and mRNA levels through immunohistochemistry (IHC) and reverse transcription polymerase chain reaction (RT-PCR), respectively. Furthermore, the correlation between LDHA expression and clinicopathological parameters was evaluated to elucidate its clinical relevance. The identification of effective diagnostic and therapeutic targets for CRC is of great importance for enhancing early detection, guiding treatment strategies, and ultimately improving patient outcomes.

## Materials and methods

Patient and clinical data collection

Four CRC tissue samples were collected to investigate the metabolism-related gene profile by a surgeon from the Division of Surgery. These tissues included two samples from early-stage (I-II) CRC and two samples from advanced-stage (III-IV) CRC. Informed consent was acquired and obtained from all participants before being enrolled in this study.

The metabolism-related gene LDHA was selected for further investigation. A retrospective assessment of LDHA expression was performed using 260 formalin-fixed, paraffin-embedded (FFPE) tissue sections collected from CRC patients diagnosed between January 2021 and December 2023 at the National Cancer Institute, Thailand.

All CRC tissue samples were histologically confirmed by the Department of Anatomical Pathology, National Cancer Institute, Thailand. Clinicopathological data collected for each patient included age at diagnosis, tumor stage, tumor size, and related parameters. Tumor staging was determined according to the TNM classification system (stages 0-IV), based on the 2017 criteria of the American Joint Committee on Cancer (AJCC), as assessed by pathologists and documented in the pathology reports.

This study was reviewed and approved by the Ethics Committee of the National Cancer Institute (approval number: 025_2021RB_IN707) based on the Declaration of Helsinki and Good Clinical Practice.

Inclusion and exclusion criteria of tissue collection

CRC tissues and matched adjacent non-tumorous tissues were obtained from patients with pathologically confirmed early- and advanced-stage CRC. All samples were collected prior to any therapeutic intervention, and patients had not received chemotherapy, radiotherapy, or targeted treatment before tissue acquisition. For the retrospective analysis, FFPE tissue specimens used for LDHA expression evaluation were retrieved from the institutional archive, comprising cases diagnosed between 2021 and 2023 with histopathological confirmation of CRC. Samples were selected based on predefined quality criteria, including tissue morphology and appropriate preservation, to ensure reliable downstream assessment.

RNA extraction and microarray analysis

Total RNA was isolated from CRC tissues and their matched adjacent normal tissues using the RNAspin Mini Kit (Cytiva, Little Chalfont, Buckinghamshire, UK) according to the manufacturer's instructions. The purity and concentration of RNA samples were assessed using a NanoDrop spectrophotometer (Thermo Fisher Scientific, Waltham, Massachusetts, United States). Gene expression profiling of metabolism-related genes was performed using microarray analysis. A total of 50 ng of RNA from each sample was labeled using the Low Input Quick Amp Labeling Kit, One-Color (Agilent p/n 5190 2305, Agilent Technologies, Santa Clara, California, United States), following the manufacturer's instructions. Briefly, 50 ng of total RNA was reverse-transcribed into double-stranded cDNA by priming with an oligo-dT primer containing a T7 RNA polymerase promoter sequence. Subsequently, in vitro transcription with T7 RNA polymerase was performed to produce cyanine 3- and cyanine 5-CTP-labeled complementary RNA (cRNA). Then, a total of 600 ng of labeled cRNA was hybridized onto an Agilent SurePrint G3 Human GE 8×60K Microarray (Design ID: 072363; Agilent Technologies, Santa Clara, California, United States) for 17 hours at 65°C with continuous rotation at 10 rpm in a hybridization oven. After hybridization, the microarray slide was washed sequentially in gene expression wash buffer 1 for one minute at room temperature and wash buffer 2 at 37°C. The arrays were then scanned using a high-resolution microarray scanner (C-model; Agilent Technologies, Santa Clara, California, United States), and raw fluorescence data were extracted from the TIFF image files using the feature extraction software (Version 12.1.0.3; Agilent Technologies, Santa Clara, California, United States) for subsequent analysis.

The expression data of CRC gene profiles in the microarray were normalized by matched adjacent normal tissues. In this study, gene expression profiles of CRC were performed using the Bioinformatics online platform (https://www.bioinformatics.com).

IHC

The expression of LDHA in cancer tissues and adjacent normal tissues was evaluated by IHC analysis. FFPE tissue sections (3 µm thick) were mounted on 3-aminopropyltriethoxysilane-coated slides. Sections of differentiated liposarcoma were used as positive controls. IHC staining was performed using a Bond-Max automated stainer (Leica Microsystems, Bannockburn, Illinois, United States). Tissue sections were deparaffinized and subjected to heat-induced epitope retrieval in Bond Epitope Retrieval Solution 2 (pH 8.9-9.1) at 100°C for 60 minutes, and a rabbit monoclonal anti-LDHA antibody (clone EPR2764Y; Cell Marque, Rocklin, California, United States; dilution 1:100) was incubated for 30 minutes. Signal detection was carried out using the Bond Polymer Refine Detection Kit (Leica Microsystems, Bannockburn, Illinois, United States) with diaminobenzidine (DAB) as the chromogen, followed by hematoxylin counterstaining. The stained sections were dehydrated, cleared in xylene, mounted with Permount, and examined microscopically for the evaluation of LDHA expression.

The IHC score was evaluated based on the percentage of positively stained cancer cells and the staining intensity. The percentage of positive cancer cells was scored as follows: 1, 0-25%; 2, 25-50%; 3, 51-75%; and 4, >75%. Staining intensity was graded as 0 for negative, 1 for weak, 2 for moderate, and 3 for strong. The final IHC score was determined by the score of positive areas × intensity scores: 0 for negative, 1-3 for weakly positive (+), 4-6 for moderately positive (++), and more than 6 for strongly positive (+++). All slides were independently evaluated by two experienced pathologists.

Quantitative real-time PCR (qRT-PCR) analysis

Total RNA was isolated from FFPE CRC tissues using the TRIzol reagent (Invitrogen, Carlsbad, California, United States) according to the manufacturer's protocol. RNA purity and concentration were assessed using a Nanodrop spectrophotometer. cDNA was synthesized from the purified RNA using the UltraScript 2.0 cDNA Synthesis Kit (PCR Biosystems Ltd, London, UK) following the manufacturer's protocol. qRT-PCR of LDHA was performed using the synthesized cDNA as a template with qPCRBIO SyGreen Mix (PCR Biosystems Ltd, London, UK). β-Actin was used as an internal control. The used primer sequences are given in Table 1.

**Table 1:** Primer sequences LDHA: lactate dehydrogenase A

Gene name	Sequences	Reference
LDHA	Forward 5′-CCCCAGAATAAGATTACAGTTGTTG-3′	[22]
	Reverse 5′-GAGCAAGTTCATCTGCCAA GTC-3′	
β-Actin	Forward 5′-GACCTTCAACACCCCAGCCCA-3′	_
	Reverse 5′-AGGCTGGAAGAGTGCCTCAG-3′	

PCR amplification was conducted under the following thermal cycling conditions: initial denaturation at 95°C for two minutes, followed by 40 cycles of denaturation at 95°C for five seconds and annealing/extension at 60°C for 30 seconds. β-Actin served as an internal reference for normalization using the real-time PCR system (Applied Biosystems, Waltham, Massachusetts, United States). Relative expression levels of LDHA mRNA were calculated using the 2^ΔΔCt^ method. All reactions were performed in triplicate.

Statistical analysis

Descriptive statistics were expressed as frequencies, percentages, means, and standard deviations (SD). Differences in gene expression among groups were analyzed using one-way analysis of variance (ANOVA). Associations between protein expression and clinicopathological parameters were evaluated using the chi-squared test. Statistical significance was defined as p<0.05 at a 95% confidence level. All statistical analyses were conducted using IBM SPSS Statistics for Windows, Version 23.0 (IBM Corp., Armonk, New York, United States) and GraphPad Prism (Dotmatics, Boston, Massachusetts, United States/Insight Venture Management, LLC, New York, New York, United States).

## Results

Cancer and adjacent normal tissues were collected from four CRC patients (age range: 53-70 years; mean±SD: 60.75±7.72 years) for metabolic gene profiling using microarray analysis. Among these, two samples were classified as early stage (I-II) and two as advanced/late stage (III-IV). Both tumor and adjacent normal tissues from each patient were analyzed, with the latter serving as controls.

**Figure 1:**
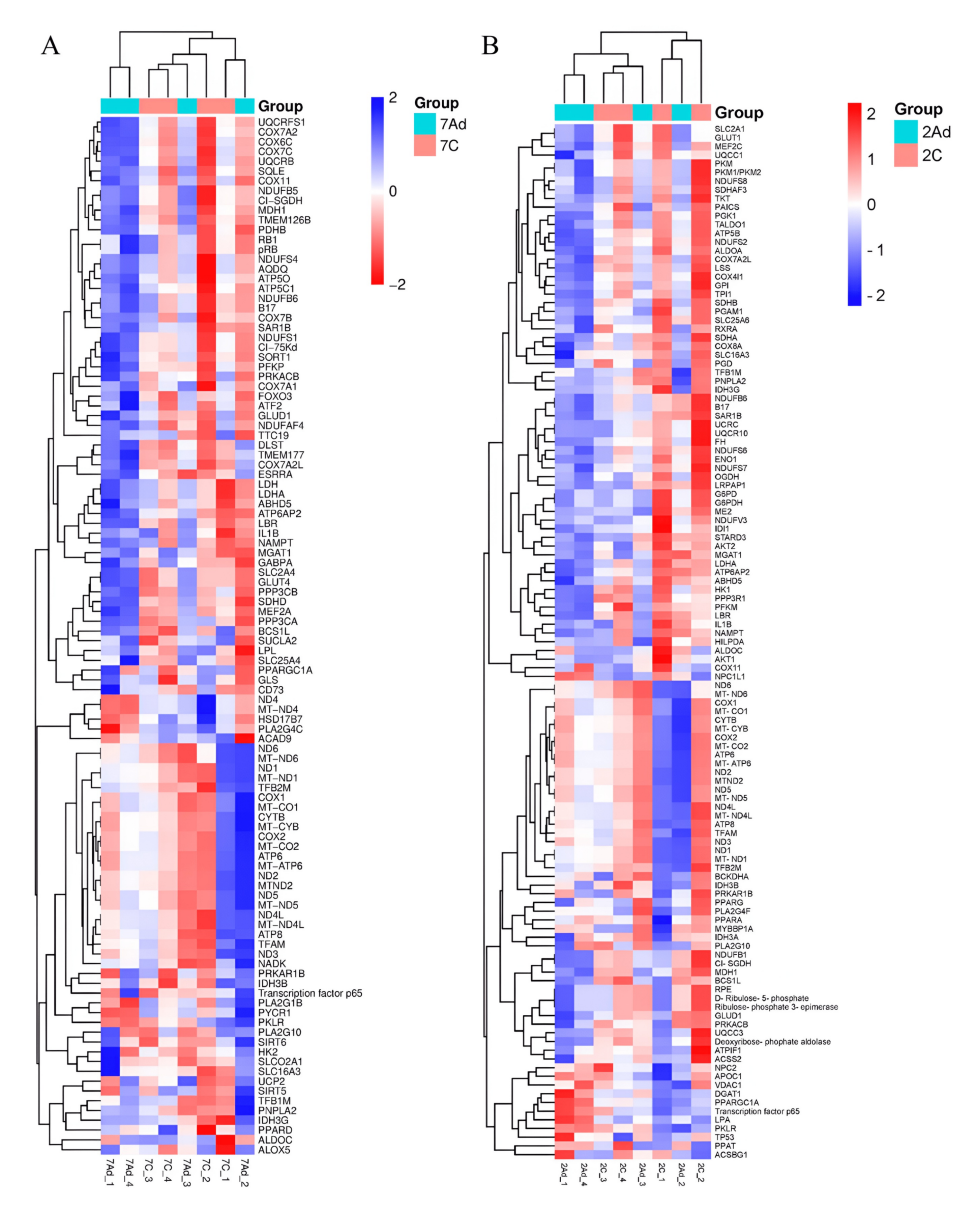
Heatmap of metabolism-related gene expression profiles in colorectal cancer Heatmap showing the expression profiles of metabolism-related genes in early-stage (A) and advanced-stage (B) colorectal cancer tissues relative to their matched adjacent normal tissues. Genes displaying more than a twofold change in expression (upregulated or downregulated) are presented.

Gene expression profiling of CRC revealed extensive metabolic reprogramming. The Kyoto Encyclopedia of Genes and Genomes (KEGG) pathway analysis and heatmap visualization were performed using the Bioinformatics online platform (https://www.bioinformatics.com) to investigate the differential expression of metabolism-related genes. The heatmap demonstrated clear differences in gene expression profiles between CRC tissues and their matched adjacent normal tissues in early and advanced stages, as shown in Figure 1.

**Figure 2:**
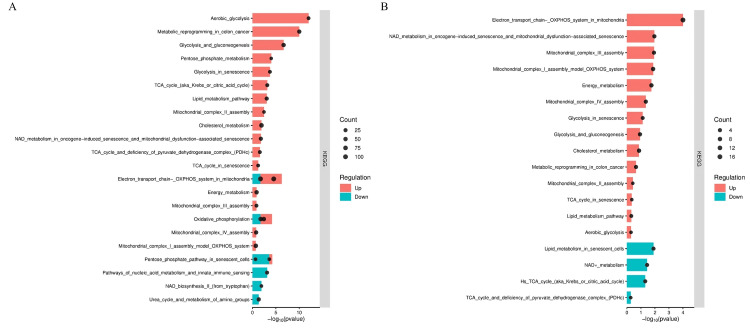
Alteration in the metabolism-related pathways of colorectal cancer Differentially expressed genes in early-stage (A) and advanced-stage (B) colorectal cancer tissues compared with their matched adjacent normal tissues were mapped to the Kyoto Encyclopedia of Genes and Genomes (KEGG) metabolic pathway system. Genes with significantly increased (upregulated) or decreased (downregulated) expression are highlighted.

Furthermore, the KEGG pathway analysis identified several significantly enriched pathways associated with CRC progression. Early-stage cancers (stage II) exhibited the upregulation of 758 pathways (13,092 genes) and the downregulation of 619 pathways (15,439 genes), while advanced-stage tumors (stage III) showed the upregulation of 630 pathways (14,655 genes) and the downregulation of 614 pathways (13,910 genes). Among these pathways, metabolism-related pathways in the early stage, notably aerobic glycolysis, glycolysis/gluconeogenesis, and CRC metabolic reprogramming, were predominantly upregulated, whereas nucleic acid metabolism and nicotinamide adenine dinucleotide (NAD) biosynthesis were downregulated. Mixed regulation was observed in pathways such as OXPHOS and the pentose phosphate pathway, underscoring complex metabolic adaptation during CRC progression (Figure 2A).

In advanced-stage CRC (stages III-IV), comparison with adjacent normal tissues revealed predominantly upregulated metabolic pathways related to energy production, including the electron transport chain, OXPHOS, glycolysis, and gluconeogenesis. Downregulation was observed in specific pathways such as lipid metabolism (Figure 2B).

From the metabolism-related profile by microarray assay, several genes were upregulated in cancer tissues, including LDHA. LDHA is a key regulatory enzyme, encoded by the LDHA gene, in the glycolytic pathway, contributing to ATP generation and supporting biosynthetic processes. It is frequently upregulated in various cancers and plays an important role in aerobic glycolysis and metabolic reprogramming, or the Warburg effect, linked to tumor progression [7,18]. Accordingly, in this study, LDHA was selected to evaluate its expression in CRC tissues. A total of 260 CRC tissue samples were collected, including 81 early-stage cases (stages 0-II; mean age 65.85±10 years) and 179 advanced-stage cases (stages III-IV; mean age 62.12±10.79 years). The majority of samples originated from the rectum (135 cases, 51.9%), followed by the sigmoid colon, retrosigmoid colon, and descending colon, respectively.

**Figure 3:**
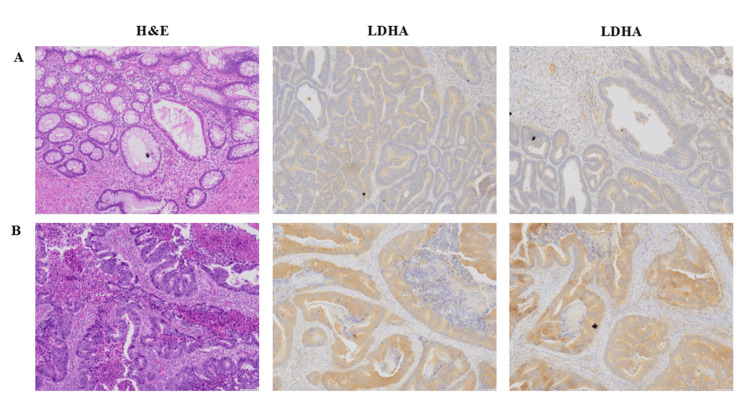
Representative LDHA protein expression in colorectal cancer tissues Specific staining with LDHA primary antibody (brown stain) is shown in colorectal cancer specimens by immunohistochemistry stain (magnification, 100×). Corresponding H&E staining of the same tissue sections. Panels represent early-stage (A) and advanced-stage (B) colorectal cancer tissues. LDHA: lactate dehydrogenase A; H&E: hematoxylin and eosin

IHC staining of CRC tissues with an LDHA-specific primary antibody demonstrated positive LDHA expression (brown staining) in both early-stage and advanced-stage cancers, as shown in Figure 3A-3B, respectively. Notably, staining intensity and the proportion of positive cells were greater in advanced-stage cancers (179/260; 68.85%) compared with early-stage cancers (81/260; 31.15%). In addition, LDHA expression appeared in both the nucleus and the membrane. Nuclear expression was more frequently observed in advanced-stage cancers. Moreover, dual localization of LDHA in both the nucleus and membrane was detected in 16/179 (8.94%) advanced-stage cases and in 4/81 (4.9%) early-stage cases. LDHA expression was also observed in normal cells in 12/260 (4.62%) cases adjacent to cancer tissues.

**Figure 4:**
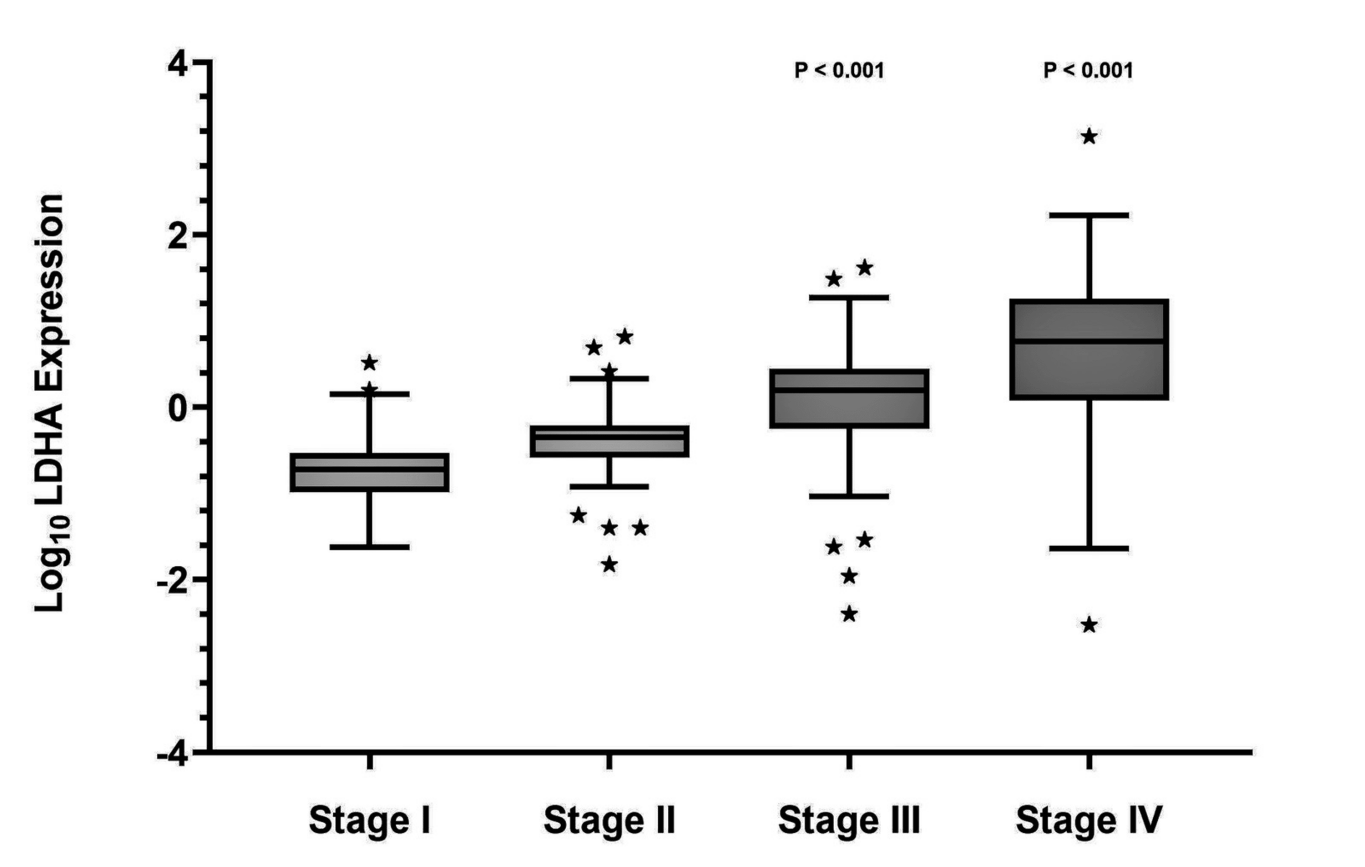
Box plot representing the expression of LDHA in colorectal cancer tissues The statistically significant association between LDHA mRNA expression in colorectal cancer specimens across different clinical stages using one-way ANOVA, in which *** means p<0.001. Bold line indicates the median values (50th percentile range) for each stage. Outliers represent extreme values. Upper asterisks indicate data points exceeding the 75th percentile range, whereas lower asterisks indicate data points falling below the 25th percentile range. LDHA: lactate dehydrogenase A; ANOVA: analysis of variance

LDHA mRNA expression in CRC samples, corresponding to those previously evaluated for LDHA protein expression by IHC, was significantly elevated in advanced-stage tumors (stages III-IV) compared with early-stage tumors (stages I-II) (p<0.001) (Figure 4).

The clinicopathological analysis of CRC cases revealed significant associations of age, disease stage, differentiation, and TNM classification with early- versus advanced-stage cancers (Table 2). High LDHA protein expression, assessed by IHC, significantly correlated with lymph node metastasis, lymphovascular invasion, and perineural invasion, but showed no correlation with sex, tumor size, or tumor location. 

**Table 2:** Association between clinicopathological parameters and LDHA protein expression in colorectal cancer tissues. LDHA expression was evaluated by IHC in a cohort of 260 colorectal cancer specimens The correlation between the clinicopathological characteristics of colorectal cancer patients and LDHA expression levels in tissue, assessed by IHC and categorized as low (<3) or high (>3), was analyzed using the chi-squared test. Statistical significance was defined as follows: *: p<0.05, **: p<0.01, and ***: p<0.001. LDHA: lactate dehydrogenase A; IHC: immunohistochemistry

Characteristic	LDHA expression		Chi square (χ²)	P-value
	Low (<3) n (%)	High (≥3) n (%)		
Age (yrs)				
≤60	66 (34.7)	35 (50)	5.016	0.025*
>60	124 (65.3)	35 (50)		
Gender				
Male	108 (56.8)	43 (61.4)	0.442	0.506
Female	82 (43.2)	27 (38.6)		
Clinical stage				
Stage 0	1 (0.5)	0 (0)	68.994	<0.001***
Stage I	31 (16.3)	1 (1.4)		
Stage II	46 (24.2)	2 (2.9)		
Stage III	84 (44.2)	22 (31.4)		
Stage IV	28 (14.7)	45 (64.3)		
T stage				
T0-T2	45 (24.2)	7 (10.8)	5.285	0.022*
T3-T4	141 (75.8)	58 (89.2)		
N stage				
N0	91 (49.5)	17 (27)	9.632	0.002**
N1-N4	93 (50.5)	46 (73)		
M stage				
M0	164 (87.7)	31 (44.9)	50.805	<0.001***
M1	23 (12.3)	38 (55.1)		
Tumor size (cm)				
≤2	29 (15.3)	9 (12.9)	0.237	0.626
>2	161 (84.7)	61 (87.1)		
Differentiate				
WD	20 (10.5)	0 (0)	11.219	0.011*
MD	159 (83.7)	64 (91.4)		
PD	3 (1.6)	4 (5.7)		
Other	8 (4.2)	2 (2.9)		
Cancer sites				
Rectum	103 (54.2)	32 (45.7)	5.549	0.593
Sigmoid colon	39 (20.5)	16 (22.9)		
Retrosigmoid colon	20 (10.5)	6 (8.6)		
Descending colon	12 (6.3)	10 (14.3)		
Transverse colon	8 (4.2)	3 (4.3)		
Cecum	4 (2.1)	1 (1.4)		
Appendix	1 (0.5)	1 (1.4)		
Other	3 (1.6)	1 (1.4)		
Lymph node metastasis				
Yes	95 (50.5)	50 (75.8)	12.689	<0.001***
No	93 (49.5)	16 (24.2)		
Metastasis				
Liver	8 (4.2)	15 (21.4)	46.296	<0.001***
Lung	5 (2.6)	4 (5.7)		
Liver and lung	5 (2.6)	11 (15.7)		
Other	1 (0.5)	3 (4.3)		
No/unknown	171 (90)	37 (52.9)		
Perineural invasion				
Presence	34 (25.4)	21 (47.7)	8.52	0.014*
Absence	11 (8.2)	1 (2.3)		
Not identified	89 (66.4)	22 (50)		
Lymphovascular invasion				
Presence	58 (33.9)	35 (60.3)	12.558	0.002**
Absence	11 (6.4)	2 (3.4)		
Not identified	102 (59.6)	21 (36.2)		
Lymph node invasion				
Presence	83 (46.1)	49 (76.6)	22.152	<0.001***
Absence	96 (53.3)	13 (20.3)		
Not identified	1 (0.6)	2 (3.1)		

## Discussion

Metabolic reprogramming is recognized as a hallmark of cancer, enabling malignant cells to adapt their bioenergetics and biosynthetic pathways to support uncontrolled proliferation [12]. In particular, aerobic glycolysis, also known as the Warburg effect, was among the most strongly activated processes in the early stages of tumorigenesis, when normal cells undergo transformation or mutation into cancer cells that require large amounts of energy to sustain rapid and continuous growth. This alteration in metabolic pathways, referred to as metabolic reprogramming, drives a shift in pyruvate metabolism from glucose-derived glycolysis toward lactate production, instead of being converted to oxaloacetate in the mitochondria [12,23]. A key driver of this metabolic shift is LDHA, which diverts pyruvate toward lactate production, thereby enhancing ATP generation and contributing to extracellular acidification. Lactate-driven acidification of the tumor microenvironment also stimulates both cancer cell and fibroblast proliferation [24,25].

From the analysis of gene expression patterns related to metabolism in CRC tissues, it was found that mechanisms associated with energy production and biosynthesis within cells, such as ATP generation through aerobic glycolysis, glycolysis, gluconeogenesis, and OXPHOS, were significantly upregulated in both the early stages (I-II) and advanced stages (III-IV) of cancer. In agreement with the findings of Knudsen et al., cancer-associated fibroblasts (CAFs) derived from primary pancreatic tumors have been shown to preferentially channel glucose metabolism toward glycolytic intermediates, supporting their survival and enhancing tumor invasiveness [26]. Similarly, Cruz et al. demonstrated that premalignant colorectal mucosa undergoes early metabolic reprogramming characterized by Warburg-like alterations and disrupted mitochondrial function. Notably, key metabolic enzymes, including PKM2, LDHA, and the glucose transporter SLC2A1 (GLUT1), were significantly upregulated in rectal mucosa from patients with precancerous lesions, further underscoring the role of metabolic shifts in early tumorigenesis [27].

In the advanced stage, aerobic glycolysis remains essential, although its activity is lower than in the early stage, where a rapid supply of energy is critical. Instead, processes such as the electron transport chain and OXPHOS were found to be more active during the advanced stage, also contributing to ATP production, albeit at a slower rate compared with aerobic glycolysis or the Warburg effect. Furthermore, evidence of metabolic reprogramming in CRC was observed through altered gene expression patterns in both early and advanced stages. Interestingly, in the early stage of CRC, some gene sets in the pathways, such as OXPHOS, exhibited either upregulation or downregulation, likely reflecting early alterations in cellular function and pathway adaptation. Moreover, these altered metabolic pathways, along with the adaptive roles of related genes and enzymes, have significant implications for the effectiveness of cancer therapy [28].

Among the genes implicated in these metabolic changes, LDHA was consistently identified across both early and advanced stages. LDHA is a metabolic enzyme that catalyzes the conversion of pyruvate to lactate. Our findings demonstrate that LDHA expression at both the protein and mRNA levels is significantly elevated in advanced-stage CRC compared with early-stage cancer tissues and adjacent normal tissues. These results are consistent with previous studies showing that LDHA overexpression is linked to poor prognosis across multiple cancers, including CRC [28] and uterine sarcoma [29]. Similarly, Cheng et al. reported elevated LDHA expression in pancreatic adenocarcinoma, where high levels of LDHA-transcribed isoenzyme-5 (LDH-5) were correlated with poor clinical outcomes. In addition, LDHA also promotes cancer cell proliferation and invasion across membranes in vitro and enhances tumor growth and metastasis in a murine pancreatic adenocarcinoma orthotopic model [22]. However, the limitations of this study were the insufficient amount of tissues and challenges in RNA purification from samples used for microarray analysis, which required multiple repeated experiments.

Moreover, LDHA protein expression was strongly associated with pathological parameters, including lymph node metastasis and distant metastases to the lung and liver, which are common in advanced CRC. These findings are consistent with those reported by Song et al., who demonstrated significantly higher expression levels of LDHA and LDHD in uterine sarcoma compared with uterine myoma. Their univariate analysis also showed that younger patients (<50 years) and those diagnosed at early stages (I-II) exhibited better prognostic outcomes than patients in advanced stages, whereas LDHA-negative patients demonstrated more favorable survival compared with LDHA-positive patients [29]. Consistent with the findings of Connell et al., CRC patients with liver metastases harboring KRAS mutations exhibited elevated LDHA expression, which was associated with reduced overall survival, highlighting the prognostic relevance of LDHA in this context [30].

## Conclusions

This study demonstrates that metabolism-related genes play a critical role in CRC progression across both early and advanced stages. Among these, LDHA emerges as a key metabolic enzyme driving reprogrammed energy metabolism to support tumor growth. LDHA is mechanistically linked to multiple cellular processes and genetic alterations associated with cancer initiation and aggressiveness. The results of this study showed that both LDHA mRNA and protein expression levels are significantly elevated in advanced-stage CRC compared with early-stage CRC. In addition, LDHA protein expression in tumor tissues was found to be associated with several clinicopathological parameters related to cancer dissemination, including cancer stage, lymphatic invasion, and metastasis to the liver or lungs. These findings reinforce the pivotal role of LDHA, not only as a critical metabolic enzyme driving tumor progression and metastasis but also as a potential biological marker for more accurate diagnosis, prognosis, and prediction of therapeutic response in CRC. The understanding of the LDHA or its downstream effects may provide novel strategies for determining effective therapeutic targets, which could be of great significance in improving clinical outcomes.
